# Correspondence

**Published:** 1871-09

**Authors:** 


### EDITORIAL CORRESPONDENCE.

CHARLIE HOPE,
BRUNSWICK COUNTY, VA., AUGUST 20, 1871.

DR. T. S. POWELL:

*Dear Doctor*—Your letter and the package, containing five numbers of THE MEDICAL COMPANION AND ADVISER, were duly received, for which, I beg you will accept my most grateful acknowledgments,—they have afforded me no small amount of pleasure and instruction. Cut off, as I have been for years, from the enjoyment and aid of the current literature of the Profession, I have felt a keen appreciation of the mental feast your kindness has given me.

I have read and examined these numbers of your journal with more than usual care,—in fact, I have given them the closest attention brought to bear upon such knowledge and experience as I have acquired during thirty years' study, and the more or less active pursuit of all matters appertaining to our Profession—more particularly its practice and its current literature. I have no hesitation in saying that THE COMPANION AND ADVISER comes nearer meeting and supplying the actual wants of the practitioner of medicine—more especially the country physician—than any publication I have yet met with. I am not prepared to say what are the wants of the city practitioner, but I feel that every country physician should be grateful and ready to acknowledge his obligation to the head which planned and the heart which supplies and makes accessible to him so large an amount of varied and practical information as is be to found upon the pages of THE COMPANION —information which he neither has time nor means of obtaining otherwise.

I feel almost assured that your enterprise will be a success. I cannot see how it can fail—although I am very well acquainted with the so called apathy of medical men—whether the charge is altogether just or not, I cannot say. My opinion is, however, that much of their indifference to the general welfare of the Profession is more apparent than real, and grows out of over-work, both mental and physical, so that much is left undone and unattempted, particularly, in collecting and recording their experience, because they have not the leisure to do full justice to their subjects, and because a large majority of us are unpracticed in literary composition. THE COMPANION kindly opens the door to all and every meritorious attempt; and I hope, through its columns to hear from many of those in the Profession, who have hitherto "made no sign," and many of whom, within my limited acquaintance, are both able and well-qualified from large and varied knowledge and experience to teach, not only the younger, but many of the older members of the Profession.

Besides the highly practical character of THE COMPANION, every true and good man must admire its tone as regards the manners and morals, or in other words, the ethics of the Profession. Just now, this is a most important subject in reference to medicine. Every thinking physician must see in the more or less distant future, and it may be the near future, that changes—radical changes must come upon everything in this unhappy country of ours—and whether or not in this progressive age, these changes will be improvements, will depend much upon the heads and hands that direct, guide, and govern medical education and opinion. In fact, from the "signs of the times," we have to fear that some of these changes (if not a number of them) will be a "let down" from the high ground we have hitherto occupied, and to you, gentlemen of the medical press, we have to look for the conservation of our high principles, and that the evil, if the evil must be, shall not be wide-spread and utterly destructive. Your journal is now taking its place in the great field of medical literature. It gives undeniable promise of great usefulness—more perhaps, than any periodical of its time—if its plan be fully, earnestly, and faithfully carried out,—and that such will be the case, none, acquainted with its editions, can doubt.

Very truly your friend, CHAS. T. LEWIS.

The above letter is from a gentleman of high character—a gentleman of superior medical and literary ability. Coming from one of the senior editor's Old Virginia friends—a companion of his boyhood and the good old times of the past—it fills his heart with pleasure and sincere gratification. It is pleasant to know that with this kind letter, from our native State and county, comes words of cheer and generous appreciation of our efforts to serve the Profession. We hope to hear from our friend and Associate Editor often, and sincerely trust the younger men of our native State—which we left 30 years ago—will take an interest in the Georgia Medical Companion, that a brother's heart may hold communion with them, and he be aided in upholding the principles of a Profession we cherish as the noblest of all—the first and last, and only object of which is the aleviation of human suffering.

